# Tele-rehabilitation: video-conferencing for delivery of interventions for people with Chronic Fatigue Syndrome

**Published:** 2012-06-15

**Authors:** Diane Cox, Heather Garry, Gavin Spickett, Louise Wilson, Chris Bojke

**Affiliations:** University of Cumbria, UK; Cumbria Partnership NHS Foundation Trust, UK; Newcastle upon Tyne Hospitals NHS Foundation Trust, UK; Northern CFS/ME Clinical Network Newcastle upon Tyne, UK; Centre for Health Economics, University of York, UK

**Keywords:** tele-rehabilitation, video-conferencing, long-term conditions, interventions, service enhancement

## Abstract

**Background:**

The delivery of services to those who are house bound or those that are unable to access transport, especially within a rural locality were identified as issues by the North Cumbria Chronic Fatigue Syndrome (CFS/ME) team. Technology that would enable the team to work with clients in their own home; without the resource implications of providing a domiciliary-based service was explored. The rationale was to provide a service to clients that have difficulty accessing clinics, particularly the severely affected and to create a more inclusive ‘closer to home’ service provision. The investigation was divided into four phases:

1. Systematic review of the literature
2. Scoping exercise via a questionnaire to all current clients
3. Feasibility and acceptability pilot with 5 clients
4. Development of larger cohort observational study

**Results:**

Phases 1–3 are complete. The review identified that in-home desktop video-conferencing is likely to be well received by patients and to be equivalent to in-person therapy in terms of clinical outcomes. Cost effectiveness of systems was shown to be reliant on initial outlay and amount of usage, and that such services could result in significant savings in time and money for patients [1]. The scoping exercise identified that there was a need for tele-rehabilitation to address the difficulties this population face in travelling to appointments, and that it was feasible as most people already had access to computers with broadband internet connection. The pilot study showed that PC-based video-conferencing in a real-life situation, linking people’s homes to a clinical base, is technically feasible and resulted in positive feedback from clients and the therapist. Technology functioned effectively, facilitating satisfactory interactions between clients and therapist. Technical problems were judged by clients and therapist not to interfere with the interaction. An implementation guide including tips for best practice and troubleshooting has been produced. This project to date has had an impact on the service and the patients. The interventions delivered by video-conferencing have resulted in high satisfaction levels for patients and have produced similar clinical outcomes to in-person delivery. This mode of delivery is being used for both consultant review sessions and patient to therapist interactions. The use of video-conferencing is now embedded into the service and offered to patients when travel or level of disability could affect attendance due to difficulties with transport or level of symptoms.

**Conclusions:**

It is feasible to use video-conferencing as a means of delivering interventions for people with chronic fatigue in rural communities. Further investigation is required to understand the differences between clinic-based and video-conferencing interventions over time.
